# Rhizoviticin is an alphaproteobacterial tailocin that mediates biocontrol of grapevine crown gall disease

**DOI:** 10.1093/ismejo/wrad003

**Published:** 2024-01-18

**Authors:** Tomoya Ishii, Natsuki Tsuchida, Niarsi Merry Hemelda, Kirara Saito, Jiyuan Bao, Megumi Watanabe, Atsushi Toyoda, Takehiro Matsubara, Mayuko Sato, Kiminori Toyooka, Nobuaki Ishihama, Ken Shirasu, Hidenori Matsui, Kazuhiro Toyoda, Yuki Ichinose, Tetsuya Hayashi, Akira Kawaguchi, Yoshiteru Noutoshi

**Affiliations:** Graduate School of Environmental, Life, Natural Science and Technology, Okayama University, Okayama 700-8530, Japan; Faculty of Agriculture, Okayama University, Okayama 700-8530, Japan; Present address: Division of Biological Science, Nara Institute of Science and Technology (NAIST), Ikoma, Nara 630-0192, Japan; Graduate School of Environmental, Life, Natural Science and Technology, Okayama University, Okayama 700-8530, Japan; Department of Biology, University of Indonesia, Depok 16424, Indonesia; Graduate School of Environmental, Life, Natural Science and Technology, Okayama University, Okayama 700-8530, Japan; Present address: Kyushu Okinawa Agricultural Research Center, National Agriculture and Food Research Organization, Miyakonojo, Miyazaki 885-0091, Japan; Graduate School of Environmental, Life, Natural Science and Technology, Okayama University, Okayama 700-8530, Japan; Graduate School of Environmental, Life, Natural Science and Technology, Okayama University, Okayama 700-8530, Japan; Department of Genomics and Evolutionary Biology, National Institute of Genetics, Mishima, Shizuoka 411-8540, Japan; Okayama University Hospital Biobank, Okayama University Hospital, Okayama 700-8558, Japan; Mass Spectrometry and Microscopy Unit, Technology Platform Division, RIKEN Center for Sustainable Resource Science, Yokohama 230-0045, Japan; Mass Spectrometry and Microscopy Unit, Technology Platform Division, RIKEN Center for Sustainable Resource Science, Yokohama 230-0045, Japan; Plant Immunity Research Group, RIKEN Center for Sustainable Resource Science, Yokohama 230-0045, Japan; Plant Immunity Research Group, RIKEN Center for Sustainable Resource Science, Yokohama 230-0045, Japan; Graduate School of Science, The University of Tokyo, Tokyo 113-8657, Japan; Graduate School of Environmental, Life, Natural Science and Technology, Okayama University, Okayama 700-8530, Japan; Faculty of Agriculture, Okayama University, Okayama 700-8530, Japan; Graduate School of Environmental, Life, Natural Science and Technology, Okayama University, Okayama 700-8530, Japan; Faculty of Agriculture, Okayama University, Okayama 700-8530, Japan; Graduate School of Environmental, Life, Natural Science and Technology, Okayama University, Okayama 700-8530, Japan; Faculty of Agriculture, Okayama University, Okayama 700-8530, Japan; Department of Bacteriology, Graduate School of Medical Sciences, Kyushu University, Fukuoka 812-8582, Japan; Western Region Agricultural Research Center (WARC), National Agricultural and Food Research Organization (NARO), Fukuyama, Hiroshima 721-8514, Japan; Graduate School of Environmental, Life, Natural Science and Technology, Okayama University, Okayama 700-8530, Japan; Faculty of Agriculture, Okayama University, Okayama 700-8530, Japan

**Keywords:** tailocin, phage tail-like bacteriocin, Allorhizobium vitris, Alphaproteobacteria, biocontrol, crown gall disease, interbacterial antagonism, grapevine

## Abstract

Tailocins are headless phage tail structures that mediate interbacterial antagonism. Although the prototypical tailocins, R- and F-pyocins, in *Pseudomonas aeruginosa*, and other predominantly R-type tailocins have been studied, their presence in *Alphaproteobacteria* remains unexplored. Here, we report the first alphaproteobacterial F-type tailocin, named rhizoviticin, as a determinant of the biocontrol activity of *Allorhizobium vitis* VAR03-1 against crown gall. Rhizoviticin is encoded by a chimeric prophage genome, one providing transcriptional regulators and the other contributing to tail formation and cell lysis, but lacking head formation genes. The rhizoviticin genome retains a nearly intact early phage region containing an integrase remnant and replication-related genes critical for downstream gene transcription, suggesting an ongoing transition of this locus from a prophage to a tailocin-coding region. Rhizoviticin is responsible for the most antagonistic activity in VAR03-1 culture supernatant against pathogenic *A. vitis* strain, and rhizoviticin deficiency resulted in a significant reduction in the antitumorigenic activity in planta. We identified the rhizoviticin-coding locus in eight additional *A. vitis* strains from diverse geographical locations, highlighting a unique survival strategy of certain Rhizobiales bacteria in the rhizosphere. These findings advance our understanding of the evolutionary dynamics of tailocins and provide a scientific foundation for employing rhizoviticin-producing strains in plant disease control.

## Introduction

Crown gall, caused by *Agrobacterium*/*Rhizobium* with tumor-inducing (Ti) plasmids, is a broad-spectrum tumorigenic disease [1]. In response to acetosyringone, a chemical released from wounded host tissues, these pathogens use virulence (*vir*) genes to transfer T-DNA containing phytohormone and opine biosynthetic genes into host cells. Once integrated into the host genome, this T-DNA leads to dedifferentiation and proliferation of the plant cells, where opines are biosynthesized as energy, carbon, and nitrogen sources for bacterial growth. Crown gall is a significant threat to grape production [2, 3], yet effective control strategies are currently lacking due to the ineffectiveness of pesticide sprays against soil-borne pathogens.

Biological control using antagonistic microorganisms represents a sustainable approach to plant disease management [2-4]. The nonpathogenic and antagonistic *Rhizobium rhizogenes* (*Agrobacterium rhizogenes*, *Agrobacterium radiobacter* biovar 2) strain K84 is a commercially available biocontrol agent for crown gall. However, its efficacy against the grapevine pathogen *Allorhizobium vitis* (*Rhizobium vitis*, *Agrobacterium vitis*) is limited [5, 6]. The *A. vitis* strain F2/5 (F2/5) was identified as an antagonist against grapevine crown gall, but its application was hampered by necrosis induction [7]. The *A. vitis* strains VAR03-1 and ARK-1, isolated in Japan, effectively suppress grapevine crown gall without inducing necrosis [2, 5, 8, 9]. They also protect roses, tomatoes, and apples from pathogenic *Rhizobium*-induced crown gall [10].

Biocontrol agents employ a variety of mechanisms, including antibiosis, competition, parasitism, induced resistance, and growth promotion in host plants. Nonpathogenic microorganisms of the same genus as the pathogens often exhibit effective biocontrol properties by antagonizing cohabiting pathogens. Interbacterial antibiosis is primarily mediated by antibiotic molecules or bacteriocins, narrow-spectrum proteinaceous, or peptide toxins. Pseudomonads have two classes of bacteriocins [11-13]. The first class includes the S-type pyocins with DNase, tRNase, rRNase, and pore-forming activities on target cells [11, 14]. Other pyocin variants, such as pyocin M with lipid II-degrading activity and bacteriocins resembling lectins or microcin-B that inhibit DNA gyrase, have also been characterized [11]. The second class includes bacteriocins with phage tail structures, classified into rigid and contractile tails (R-type) and flexible and noncontractile tails (F-type), represented by the prototypical R-pyocins and F-pyocins of *Pseudomonas aeruginosa*, respectively [11-13, 15]. These tailocins are thought to have evolved from bacteriophages and function by forming pores in the target bacterial membrane, leading to inhibition of protein and nucleic acid synthesis and cell death [16]. Bacteriocinogeny also involves the injection of toxic effector proteins into target cells via a secretion system [11, 17, 18].

The biocontrol activity of *Rhizobium rhizogenes* K84 is mainly based on agrocin 84, an adenine nucleotide analog that inhibits RNA, DNA, and protein synthesis, along with amino acid transport, ultimately killing the pathogens [19]. Similarly, F2/5 produces agrocin-like antibiotics; however, its antibiosis mechanism appears to be dependent on antibiotic peptide(s) [7, 20]. In previous work, we demonstrated that *A. vitis* VAR03-1 (VAR03-1) exhibited growth inhibitory activity against the pathogenic *A. vitis* strain VAT03-9 (Ti) (VAT03-9 (Ti))*.* This inhibitory effect was observed in the culture filtrate, and the active substance(s) responsible for the biocontrol activity were identified as heat-labile large molecules (>100 kDa) [21].

In this study, we present a newly discovered F-type tailocin, designated as rhizoviticin, as the key factor for the antagonistic activity of VAR03-1 against crown gall. It is responsible for the antibiotic effect of VAR03-1 culture filtrate on pathogenic *A. vitis*. To the best of our knowledge, this is the first discovery of a tailocin within the *Alphaproteobacteria* class that is widely distributed in *A. vitis* strains. Our study also sheds light on the intriguing feature of the rhizoviticin-coding locus, which represents an early evolutionary intermediate in the transition from a prophage to a tailocin-coding region.

## Materials and methods

### Bacterial strains, media, and growth conditions

The *A. vitis* strains VAT03-9 (Ti), VAR03-1, and VAR06-30 were cultured in King’s B (KB) medium at 27°C [5]. For growth assays using culture filtrate, a previously described method was used [21]. Briefly, precultured *A. vitis* strains and mutants were inoculated into 15 ml KB (OD600 of 0.2) and incubated with shaking for 24 h. Culture filtrate was then prepared by centrifugation (4000 rpm, 40 min) and filtration (0.45 μm pore size membrane, Millex-HA, Merck). Cell suspensions of VAT03-9 (Ti) (OD600 of 0.4) were inoculated into each culture filtrate (final OD600 of 0.1) with or without 30 μM acetosyringone (Sigma-Aldrich) and incubated at 27°C with shaking.

### Ribonucleic acid extraction and transcriptional analysis

Bacterial ribonucleic acids (RNAs) were extracted using the PureLink RNA Mini Kit (Thermo Fisher Scientific), and cDNAs were synthesized using the PrimeScript RT Reagent Kit with gDNA Eraser (Takara Bio). Relative *virE2* expression levels were quantified by qRT-PCR using the Luna Universal qPCR Master Mix (New England Biolabs) and the LightCycler 96 system (Roche) with *pyrG* as a control [22].

### Ultraviolet irradiation for growth inhibition assay and transcriptional analysis

Strains VAT03-9 (Ti), VAR03-1, and rhizoviticin-deficient mutants were inoculated (OD600 of 0.1) into 15 ml KB to reach an OD600 of 0.5. After exposure to ultraviolet (UV) (302 nm) for 30 s using a ChemiDoc XRS+ trans-illuminator (Bio-Rad), they were further incubated for 24 h with shaking. The culture filtrates were used for the growth inhibition assay with VAT03-9 (Ti). For gene expression analysis (*recA*, *22810*, and *22960*), VAR03-1 was inoculated into 15 ml KB (OD600 of 0.3) and cultured until OD600 reached 0.9. After UV exposure, 2 ml of each culture was sampled at 1 and 3 h. RNA extraction was followed by relative gene expression measurement using qRT-PCR with specific primers (Supplementary Table S1). To analyze selected genes in rhizoviticin-deficient mutants, transcript copy numbers were quantified by the absolute quantification method using a synthesized DNA fragment (1261 bp) with tandem amplificon sequences of each target gene and specific primers (Supplementary Table S1) to create a standard curve. Relative copy numbers were normalized to the amount of template cDNA and expressed as copy numbers per 25 ng RNA used for cDNA synthesis.

### Purification of rhizoviticin

VAR03-1 (OD600 of 0.1) was cultured in 100 ml KB in a 300 ml Erlenmeyer flask with shaking until OD600 reached 0.5. The culture was then transferred to a sterile 50 ml conical tube and exposed to UV (30 s). It was subsequently returned to the flasks and incubated for 24 h. Tailocin purification was performed according to a reported method [23]. Benzonase nuclease (10 μl, 25 U/μl) (Sigma-Aldrich) was added to the culture and incubated (30 min, 37°C, gentle shaking). The cultures were centrifuged (8000 × *g*, 30 min, 4°C), and the supernatants were filtrated (0.22 μm pore size membrane, Millex-GS, Merck). The filtrate was combined with an equal volume of polyethylene glycol (PEG) solution (20% PEG 6000, 1.0 M NaCl) and incubated overnight at 4°C with agitation (200 rpm). The suspension was centrifuged (8000 × *g*, 1 h, 4°C). The precipitates were dissolved in 10 ml TN50 buffer (10 mM Tris HCl, pH 7.5, 50 mM NaCl) and centrifuged (4000 × *g*, 15 min, 4°C). The supernatants underwent ultracentrifugation (48 400 × *g*, 4°C, 3 h; P55ST2 rotor, GP56GII, HITACHI). The precipitates were resuspended in 2 ml TN50 buffer, filtered with Millex-HA, and fractionated by gel filtration on an AKTA pure 25 with a HiPrep 16/60 Sephacryl S-500 HR column (GE Healthcare). The bactericidal activity of each fraction was evaluated using a spot assay on KB soft agar containing VAT03-9 (Ti). One fraction and the original precipitate suspension were concentrated using a centrifugal filter unit (Amicon Ultra, 0.5 ml, 100 kDa, Merck) and examined by transmission electron microscopy (TEM).

### Transmission electron microscopy

Copper grids were coated with plasma-polymerized naphthalene film (Super support film, Nisshin EM) and hydrophilized by glow discharge. Two-microliter fractions were pipetted onto the grids and incubated for 60 s. Excess solution was removed by blotting with filter paper. The grids were then incubated twice for 10 s in 2% uranyl acetate solution and blotted again. After drying, the samples were examined using a JEM-1400Flash transmission electron microscope (JEOL) at 80 kV with sCMOS camera. The length of the phage tail-like structures was measured using ImageJ (version: 2.0.0.-rc-69/1.52p).

### Transposon-mediated random mutagenesis

A spontaneous VAR03-1 nalidixic acid (NA)-resistant mutant (VAR03-1 (nal)) was obtained by selection on NA (30 μg/ml)-containing KB agar. *Escherichia coli* S17-1 carrying the transposon (Tn)-harboring vector pBSLC1 [24] and VAR03-1 (nal) were cultured in KB liquid medium with kanamycin (KM) and NA, respectively, overnight. Cells were collected by centrifugation, resuspended in fresh KB, and mixed thoroughly. Cells were again collected by centrifugation and resuspended in fresh KB. This suspension was spotted onto a sterilized mixed cellulose esters membrane (0.22 μm, 13 mm, Millipore) on KB agar and incubated overnight at 27°C. The membrane was transferred to KB liquid medium. After vortexing, an aliquot was plated on KB agar with KM and NA, and mutant colonies were obtained after overnight incubation at 27°C.

### Screening for antagonism-deficient mutants

Tn-insertion mutants of VAR03-1 (nal) were transferred to KB agar containing KM and NA as well as to antibiotic-free KB agar. After colony formation by incubation, a cell suspension of VAT03-9 (Ti) in KB soft agar was overlaid on the antibiotic-free KB agar. Colonies without halos were selected and picked up from the duplicate plate.

### Identification of Tn-insertion sites

The Tn-insertion sites of the mutants were identified according to a previously described method [24].

### Deletion of targeted genes

Each target gene in VAT03-1, flanked by 600 bp sequences, was PCR amplified with specific primers (Supplementary Table S1), cloned into pGEM-T Easy vector (Promega), and subjected to inverse PCR using primers with BamHI or HindIII sites (Supplementary Table S1) outside the coding region. After purification and restriction enzyme digestion, self-ligation and transformation into *E. coli* DH5α were performed. Deletion confirmation by sequencing was preceded by excision of the insert fragment (for *22790*, *22800*, and *22810*) using EcoRI and recombination into the EcoRI site of the pK18mobsacB vector. For *23000*, *23010*, *23030*, *23030*, and *23040*, insert DNA and the pK18mobsacB vector were coamplified with primers containing 15 bp overlapping sequences and ligated using the In-Fusion HD Cloning Kit (Takara Bio). In the case of *22970*, the plasmid resulting from digestion with PstI and subsequent blunting of 3′-overhangs with T4 DNA polymerase (Takara Bio) was cloned into the vector between the SphI and SmaI sites after further digestion with SphI. These constructs were transformed into *E. coli* S17-1 *λpir*, and the plasmids were then conjugally transferred into VAR03-1 (nal). VAR03-1 (nal) colonies, expected to contain both the wild-type (WT) and deleted forms of the target gene due to homologous recombination, were suspended in fresh KB, plated on KB agar containing sucrose (10% w/v) and NA, and surviving colonies lacking the *sacB* gene from the second homologous recombination were screened, with selected colonies carrying the deleted form of the target gene validated by colony PCR.

### Evaluation of the bacterial tumor-suppressing activities on tomato and grapevine

Bacterial cells were harvested from overnight cultures by centrifugation, resuspended in fresh KB (OD600 of 0.2), and applied to the stems of 3-week-old tomato (*Lycopersicon esculentum* Mill. cv. Ponderosa) or 6-month-old grapevine (*Vitis vinifera* L. cv. Neo Muscat) plants using sterilized wooden toothpicks. Subsequently, cell suspensions of other bacterial strains (VAR06-30, VAR03-1 or mutants) were inoculated at the same site, with sterilized distilled water as a negative control. After 4-weeks of incubation, symptom severity was scored on a 0–4 gall size scale, where 0 = none, 1 = very small, 2 = small, 3 = medium, and 4 = large. Results were expressed as a percentage of the gall size rate.

## Results

### Isolation of the antagonism-deficient VAR03-1 mutants

**Figure 1:**
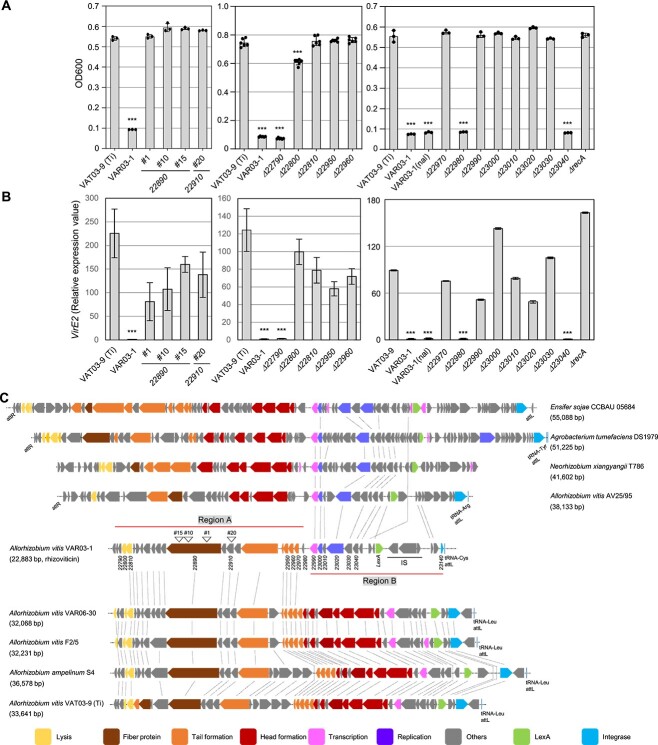
Interbacterial antagonistic activities of *A. vitis* VAR03-1 and its rhizoviticin-deficient mutants against *A. vitis* VAT03-9 (Ti) and the genome organizations of the rhizoviticin-coding region and related prophages. Growth (A) and relative expression levels of *virE2* (B) of VAT03-9 (Ti) in the supernatants prepared from the bacterial culture of VAT03-9 (Ti), VAR03-1, and its mutants; values represent OD600 (A) or relative expression level of *virE2* (B) after 24 h growth of each culture; each bacterial strain was cultured for 24 h in liquid medium, and supernatants were prepared by centrifugation and filtration; VAT03-9 (Ti) was then inoculated (initial OD600 of 0.1) and grown in the supernatant-supplemented medium with acetosyringone; data are presented as means, and error bars represent SD (*n* = 3 for the left and right panels, *n* = 6 for the middle panel); asterisks indicate statistically significant differences from control (one-way ANOVA with Dunnett’s *post hoc* test, ^*^^*^^*^P < .0001); experiments were repeated three times, and representative results are shown; (C) schematic representation of the genetic organization of the rhizoviticin-coding genomic region in VAR03-1 (AP023268.1) and the related prophages in *A. vitis* VAT03-9 (Ti) (AP023279.1), VAR06-30 (AP023272.1), F2/5 (CP055268.1), AV25/95 (JAALYG010000006.1), *A. ampelinum* S4 (CP000633.1), *Neorhizobium xiangyangii* T786 (JAJAWH010000032.1), *Agrobacterium tumefaciens* DS1979 (JAGIPE010000002.1), and *Ensifer sojae* CCBAU 05684 (CP023067.1); each gene is represented by arrows with colors based on their potential functions. The positions of Tn-insertion in each mutant used in (A) and (B) are shown as inverted triangles; dotted lines indicate homologous genes (see Table S3 for the e-values and % sequence identities of the genes in the rhizoviticin-coding genomic region to the corresponding genes of VAR06-30 and VA25/95 prophages).

To elucidate the genetic determinants underlying the antagonistic activity of VAR03-1 against VAT03-9 (Ti), we performed Tn-mediated random mutagenesis. Mutant colonies were grown on replicated agar plates, with one plate overlaid with soft agar containing VAT03-9 (Ti). By screening of ~4000 colonies, we obtained four mutants (#1, #10, #15, and #20) that failed to produce halos (Supplementary Fig. S1). The culture filtrates of these mutants neither suppressed the growth of VAT03-9 (Ti) nor induced the acetosyringone-mediated *virE2* expression (Fig. 1A and B), indicating a loss of *in vitro* antagonistic activity.

We then determined the Tn-insertion sites in each VAR03-1 mutant using the plasmid rescue method. Three mutants (#1, #10, and #15) independently carried Tn-insertions within a homolog of the phage tail fiber gene from *Allorhizobium ampelinum* S4 (S4), a pathogenic strain of grapevine crown gall in Hungary [25] (Fig. 1C). In the remaining mutant (#20), the Tn insertion was found in a homolog of an uncharacterized gene from S4 located upstream of the phage tail fiber gene (Fig. 1C). These results suggest a possible link between the antagonistic activity of VAR03-1 and a prophage-like (PPL) element.

### Headless prophage-like region underlies interbacterial antagonism of VAR03-1

To investigate the genomic organization surrounding the genes responsible for the antagonistic activity of VAR03-1, we performed whole genome sequencing (WGS) of VAR03-1, VAT0-9 (Ti), and *A. vitis* VAR06-30 (VAR06-30; a nonpathogenic and nonantagonistic strain) for comparison [26-28]. Using PHASTER analysis with the S4 genome, we identified a total of 3, 5, 6, and 6 prophage or PPL regions on the large chromosome (Chr 1) of VAR03-1, VAT03-9 (Ti), VAR06-30, and S4, respectively (Supplementary Fig. S2A–D). Further analysis revealed that the Tn-insertion-disrupted genes in the VAR03-1 mutants (IDs; *22890* and *22910*) were both located within a PPL region (Fig. 1C and Region 2 in Supplementary Fig. S2A). Similarly, VAR06-30, S4, and VAT03-9 (Ti) also possessed homologs of these genes within potentially intact prophages integrated into a tRNA-Leu gene in each strain (Fig. 1C and Supplementary Fig S2B–D; additional details provided later). A comparable prophage was also identified at the tRNA-Leu gene locus in F2/5, with a length of 30–35 kb (Fig. 1C). In contrast, the PPL region of VAR03-1 was relatively smaller, spanning 22.7 kb (Fig. 1C). Its similarity to these prophages was restricted to a 14.6 kb subregion (termed Region A), which encompassed genes associated with tail formation and cell lysis. Notably, genes responsible for head formation and other functions present in the VAR06-30, F2/5, S4, and VAT03-9 (Ti) prophages were absent from the VAR03-1 PPL region (Fig. 1C and Supplementary Table S2). Instead, the PPL region contained a distinct subregion (termed Region B) that harbored genes homologous to prophage genes identified in several bacteria, including *A. vitis*, *Neorhizobium xiangyangii*, *Agrobacterium tumefaciens*, and *Ensifer sojae* (Fig. 1C). These genes were located upstream of the morphogenic gene clusters in each prophage, with a total genomic length of 32–43 kb. Region B of VAR03-1 contained genes encoding the helix-turn-helix domain of a DnaA protein (IDs; 23000), a DnaB-like replicative DNA helicase (23020), a crossover junction endodeoxyribonuclease RuvC (23030), a LexA family transcriptional repressor protein (23080), and six uncharacterized proteins (Supplementary Table S2), but no genes related to head formation. These results suggest that the PPL region of VAR03-1 is a chimera of two different types of phage genomes. Furthermore, the absence of head formation genes strongly suggests that this region may encode a tailocin [11, 15].

To evaluate the involvement of the PPL region of VAR03-1 in its antagonistic activity, we generated deletion mutants targeting five genes within Region A or its adjacent left flanking region (*22790*, *22800*, *22810*, *22950*, and *22960*). These genes encode a hypothetical protein, a membrane protein, a chitinase, the phage tail tube protein GTA-gp10, and a phage tail tube protein, respectively (Fig. 1C and Supplementary Table S2). Except for the *Δ22790* mutant, the other four mutants showed either complete or partial loss of their growth inhibitory activity against VAT03-9 (Ti) (Fig. 1A) and reduced suppression of *virE2* expression (Fig. 1B). These results suggest that the genes in Region A are essential for interbacterial antagonism. The gene product of *22800* contains a membrane-binding domain, implying a potential holin-like function that facilitates access of a lytic enzyme, probably encoded by *22810*, to the peptidoglycan, thereby inducing cell lysis [29].

We further probed the genetic basis of the antagonistic activity of VAR03-1 by generating and analyzing deletion mutants for two Region A genes (*22970* and *22980*; encoding a DUF3168 domain-containing protein and an ssDNA-specific exonuclease, respectively) and six Region B genes (*22990*, *23000*, *23010*, *23020*, *23030*, and *23040*; encoding an RNA polymerase sigma-C factor, a helix-turn-helix domain DnaA protein, a hypothetical protein, a DnaB-like replicative DNA helicase, a crossover junction endodeoxyribonuclease RuvC, and a putative DNA binding protein, respectively). The growth inhibitory activity was abolished in six mutants, except for *Δ22980* and *Δ23040* (Fig. 1A), and they exhibited reduced suppression of *virE2* expression (Fig. 1B). Collectively, these results indicate that both Region A and Region B genes play an essential role in the antagonistic activity of VAR03-1.

### SOS-dependent induction of interbacterial antagonism and prophage-like genes in VAR03-1

**Figure 2:**
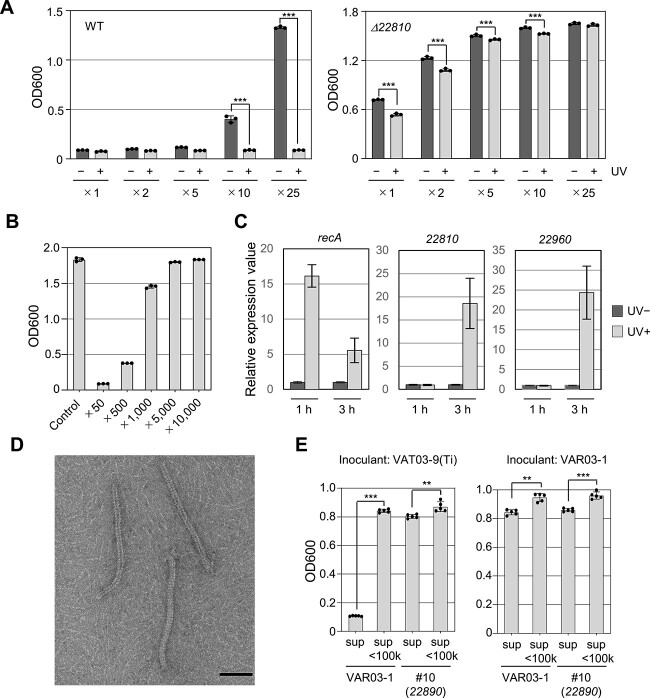
UV-induced enhancement of rhizoviticin-dependent antagonistic activities of *A. vitis* VAR03-1 and electron micrograph of rhizoviticin; (A) growth inhibitory activities of the diluted filtrates prepared from the cultures of VAR03-1 (left) and its rhizoviticin-deficient mutant *Δ22810* (right) with or without UV irradiation. Values represent the OD600 of each culture after 24 h of growth. Each bacterium was grown for 24 h in liquid medium, and supernatants were prepared by centrifugation and filtration; VAT03-9 (Ti) was then inoculated (initial OD600 of 0.1) and grown in supernatants serially diluted with fresh medium as indicated; data are presented as mean values and error bars represent SD (*n* = 3); asterisks indicate statistically significant differences from control (two-way ANOVA with Sidak’s *post hoc* test, *P* < .0001); (B) growth suppressive activities of the filtrates of UV-irradiated VAR03-1 culture at higher order dilutions; (C) relative expression levels of *recA*, *22810*, and *22960*; the VAR03-1 cells were grown in liquid medium (initial OD600 of 0.1) for 24 h and UV irradiated; cells were harvested from the cultures at 1 and 3 h after UV exposure and used for RNA preparation; as a control, the VAR03-1 cells were cultured without UV exposure; gene expression levels of each sample were measured by qRT-PCR using the relative quantification method; data are presented as mean values and error bars represent SE (*n* = 3); (D) electron microscopic structure of rhizoviticin in the concentrated fraction of VAR03-1 supernatants showing the highest antibiotic activity against VAT03-9 (Ti). Flexible phage tail-like structures are observed; the mean size of rhizoviticin is 164.18 ± 24.48 nm in length and 8.84 ± 0.69 nm in width (SD, *n* = 49); bar = 50 nm; (E) growth suppressive activities of the size (100 kDa)-fractionated culture filtrate of WT and rhizoviticin-deficient mutant of VAR03-1 against VAT03-9 (Ti) and VAR03-1 itself.

To test the inducibility of antagonistic activity mediated by the PPL region in VAR03-1, we exposed VAR03-1 cells to UV irradiation. UV exposure and genotoxic stressors like mitomycin C can trigger the SOS response, leading to tailocin production similar to prophage induction [30-32]. The culture filtrate collected 24 h after UV-irradiation showed significantly higher growth inhibitory activity against VAT03-9 (Ti) than nonirradiated cells. Complete growth inhibition was observed at a 25-fold dilution for the UV-irradiated culture (Fig. 2A), with detectable inhibitory activity even at a 500-fold dilution (Fig. 2B). Consequently, the antagonistic activity of VAR03-1 was remarkably enhanced, showing a 40–200-fold increase upon UV irradiation. In contrast, the culture filtrate of the *Δ22810* mutant showed weaker growth inhibition of VAT03-9 (Ti) compared to the WT. Moreover, slight but significant differences in inhibitory activity were observed between the UV-treated and untreated cultures of the *Δ22810* mutant (Fig. 2A).

To validate the involvement of the SOS response in enhancing the UV-induced antagonistic activity, we generated a *recA* deletion mutant (*ΔrecA*) (RvVAR031_21770) in VAR03-1. The *ΔrecA* mutant showed a significant loss of growth inhibition against VAT03-9 (Ti) (Fig. 1A) and an increase (not suppression) in *virE2* expression (Fig. 1B), even without UV irradiation. These results suggest that the antagonistic activity of VAR03-1 depends on *recA*, most likely via the SOS response.

We hypothesized that a LexA-like protein (23080) functions as a temperate phage CI repressor (Fig. 1C) and that its autodegradation, facilitated by activated RecA protein, contributes to the antagonism expression. To investigate this, we analyzed *recA* expression in VAR03-1 after UV irradiation and confirmed its upregulation at 1 h postirradiation (Fig. 2C). Further analysis revealed that the expressions of *22810* and *22960* in the PPL region were induced at 3 h (not at 1 h) after UV irradiation (Fig. 2C). These results support the idea that the synthesis of the active substance responsible for the antagonistic activity encoded by the PPL region is inducible, similar to the production of tailocin.

### The prophage-like region encodes the flexible-type phage tail structure

To visualize the active substance responsible for the antagonistic activity, we subjected the UV-irradiated VAR03-1 liquid culture filtrate to ultracentrifugation, followed by separation of the precipitate suspension by gel filtration chromatography (Supplementary Fig. S3A). The most potent fraction was subsequently analyzed by TEM (Fig. 2D and Supplementary S3B). TEM imaging revealed the presence of a single type of macromolecule resembling phage tails in the fraction. This structure, estimated to be 164.18 nm in length and 8.84 nm in diameter, belongs to the flexible type, which lacks visible tail fibers under our experimental conditions. These results suggest that the substance responsible for the antagonistic activity of the VAR03-1 supernatant is a tailocin, which we have designated as rhizoviticin. Although the VAR03-1 genome features two PPL regions in Chr1 and one in Chr 2, they all contain genes associated with head formation (Supplementary Fig. S2E). Since we did not detect headed phage-like particles in this active fraction, it is likely that rhizoviticin primarily contributes to the antagonism. We confirmed that the bactericidal activity observed in the culture filtrate of VAR03-1 against VAT03-9 (Ti) does not exhibit self-replication. This was validated by assessing the killing activity of the supernatant of an overnight culture of VAT03-9 (Ti) after inoculation with concentrated and size-fractionated VAR03-1 supernatant (Supplementary Fig. S4).

**Figure 3:**
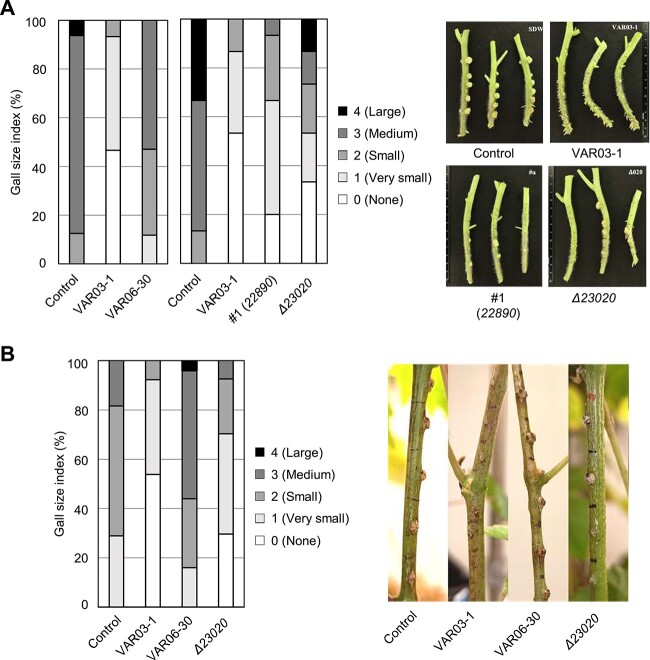
Reduction of antitumorigenesis activity of rhizoviticin mutants; disease index (left) and tumor formation (right) on tomato (A) and grapevine (B) after inoculation of *A. vitis* VAT03-9 (Ti) with *A. vitis* VAR06-30, VAR03-1, or rhizoviticin-deficient mutants of VAR03-1 (#1; Tn-insertion mutant of *22890* and *Δ23020*; a deletion mutant of 23 020 in Region A); the indicated bacterial strains and mutants were grown in liquid medium and the cultures, whose OD600 was adjusted to 0.1 with fresh medium, were coinoculated with VAT03-1 (Ti) on tomato or grapevine stems using toothpicks; after 3 weeks, tumors formed at eight inoculation points were classified into five categories according to size as indicated, and the proportion of each category was presented as a disease index for each bacterial strain; experiments were repeated three times, and representative results are shown.

### Contribution of rhizoviticin to the antitumorigenic activity in plants

We investigated whether the in planta biocontrol activity of VAR03-1 is dependent on rhizoviticin [5]. Two rhizoviticin-deficient mutants, #1 (Tn-insertion in *22890*) and *Δ23020*, were selected as representative mutants of the genes in Regions A and B, respectively, and their suppressive activities on gall formation by VAT03-9 (Ti) were examined [5]. Although tumors developed at the site inoculated with the pathogen alone, coinoculation with VAR03-1 effectively suppressed tumor formation, in contrast to VAR06-30 (Fig. 3A). Conversely, coinoculation with the two rhizoviticin-deficient mutants increased tumor formation compared to the WT, indicating reduced antitumorigenic activities (Fig. 3A).

To further evaluate the correlation between *in vitro* antibiotic activity and in planta tumor suppression, we performed similar experiments with the deletion mutants *Δ22790*, *Δ22800*, *Δ22810*, *Δ22950*, and *Δ22960*. Their tumor-suppressive activities on tomato plants were correlated with their *in vitro* antibiotic activities, as shown in Fig. 1A and B (Supplementary Fig. S5). A significant reduction in disease suppression was observed only in the case of the four mutants, *Δ22800*, *Δ22810*, *Δ22950*, and *Δ22960*, which exhibited diminished *in vitro* antibiotic activities. Additionally, we examined the disease-suppressing activity of the rhizoviticin-deficient mutant *Δ23020* on grapevine (Fig. 3B). In this system, VAR03-1 effectively suppressed gall formation, whereas VAR06-30 did not. Although the *Δ23020* mutant showed some disease suppression, its efficacy was weaker compared to the WT. These results support the critical role of rhizoviticin in the biocontrol activity of VAR03-1 against crown gall.

In this series of experiments, the rhizoviticin-deficient mutants displayed residual antagonistic activity in tumor suppression in plants, despite a remarkable reduction in interbacterial antibiotic activity *in vitro* (Fig. 1A and B). Correspondingly, the culture filtrate from *Δ22810* exhibited a reduced growth-suppressive effect against VAT03-9 (Ti) (Fig. 2A). To investigate the nature of this residual activity, we compared the antibiotic activity of the culture filtrate from #10 (Tn-insertion in *22890*) with and without size fractionation (100 kDa cutoff). The high-molecular-weight fraction showed a weak antibiotic activity against VAT03-9 (Ti) (Fig. 2E), suggesting the presence of other high-molecular-weight substance(s) with antibiotic activity produced by VAR03-1 (Supplementary Fig. S2A and E). These substance(s) could contribute to the gall-suppressing activity of the mutant. One potential candidate for this residual activity is the prophage(s) integrated into the VAR03-1 genome. However, it is important to note that the high-molecular-weight fraction also showed a weak self-inhibitory activity (Fig. 2E) [21], which was also observed in the culture filtrate of the rhizoviticin-deficient mutant. Hence, the involvement of prophages in the residual activity seems unlikely, and the precise identification of the responsible substances remains elusive.

### Rhizoviticin is a possible early evolutionary tailocin

The genetic locus governing rhizoviticin production comprises two regions originated from evolutionarily distinct sources (Fig. 1C). Prophages containing a genomic segment highly similar to Region A were identified in at least four *A. vitis* strains (Fig. 1C) and integrated into the same chromosomal position within the tRNA-Leu gene. These prophages appeared to be intact and contained a complete set of phage genes (Fig. 1C, Supplementary Fig. S6B and Table S3). Prophages carrying a segment homologous to Region B were found in several bacteria, including an *A. vitis* strain. Two of them appeared to be intact and integrated into tRNA genes (tRNA-Arg in *A. vitis* AV25/95 and tRNA-Tyr in *A. tumefaciens* DS1979; Fig. 1C, Supplementary Fig. S6C–E and Table S3). Genes homologous to those in Region B were also identified in other *A. vitis* strains with registered WGS data, but their prophage genome organizations could not be analyzed due to their incomplete assembly status.

Loci resembling the rhizoviticin-coding region were found in eight *A. vitis* strains from various countries (Supplementary Fig. S7). All these loci occupied the same genomic region immediately downstream of a tRNA-Cys gene on Chr 1 (Supplementary Fig. S6A). Although minor variations, like IS insertions, were observed, this situation implies a broad geographic distribution of rhizoviticin-producing *A. vitis* strains. A more in-depth analysis of the region beyond the tRNA gene revealed a truncated integrase gene (*int*), suggesting that the right lateral boundary of the rhizoviticin locus lies between the *int* and the tRNA gene. Although no duplicated sequences corresponding to the *attL*/*R* sites were detected, the left boundary between *22750* and *22760* was delineated by the sequence comparison of the left end of Region A with the corresponding genomic region of *A. vitis* VAT06-30 (lacking the rhizoviticin-related genomic region) (Supplementary Fig. S6A). These genomic features suggest that the ancestral prophage integrated into the tRNA-Cys gene, and subsequent gene deletions for head formation and other functions, such as integration/excision, contributed to the transformation of this locus into a tailocin-coding locus. A distinctive characteristic of the rhizoviticin genomic region as a tailocin-coding locus is the presence of genes apparently related to replication, typically found in the early regions of phage genomes but not in the other tailocin-coding regions. Furthermore, five genes in Region B were found to be indispensable for rhizoviticin-mediated interbacterial antagonism (Figs 1A and B and 3). The presence of these genes, together with a remnant of the integrase gene, suggests that the rhizoviticin-coding locus may represent an early stage of evolution from a prophage to a tailocin.

### Role of genes in Region B in rhizoviticin production

**Figure 4:**
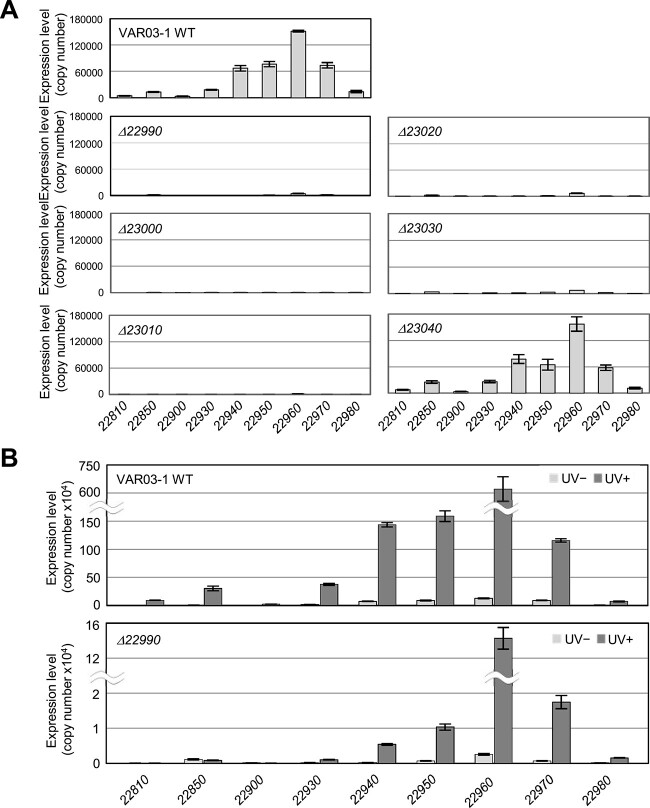
Functions of the genes in the rhizoviticin-coding locus: Roles of genes in Region B in the expression of genes in Region A; (A) transcript levels of the selected genes in Region A, which are required for rhizoviticin biosynthesis and cell lysis in WT and deletion mutants of six genes in Region B; (B) transcript levels of the selected genes in Region A in WT and a deletion mutant (*Δ22990*) with or without UV irradiation; cells of WT VAR03-1 or the mutants were grown in liquid medium (initial OD600 of 0.3) and irradiated with UV when the OD600 reached 0.9; the cells were then cultured for 3 h and used for RNA preparation; as a control, the cells were cultured without UV irradiation; gene expression levels of each sample were measured by qRT-PCR using the absolute quantification method; data are presented as mean values, and error bars represent SE (*n* = 3).

A homology search revealed that numerous Region A genes are associated with tail formation, namely rhizoviticin biosynthesis, and cell lysis (Supplementary Table S2). However, the functions of Region B genes remain unclear, although some may be involved in regulating the expression of Region A genes. To gain further insight, we constructed deletion mutants targeting Region B genes (*22990*, *23000*, *23010, 23020*, *23030*, *2340*, and *23080*) and analyzed the expression of selected Region A genes (*22810*, *22850*, *22900*, *22930*, *22940*, *22950*, *22060*, and *22970*) in these mutants (Fig. 4A). Of the seven genes in Region B that we attempted to delete, we were unable to generate a deletion mutant for *23080*, which encodes a LexA family repressor. This limitation may be due to the induction of cell lysis genes in the rhizoviticin-coding locus. It also suggests that 23080 probably acts as the major repressor of this locus, analogous to the CI repressors found in many temperate phages.

Deletion of the remaining six genes in Region B significantly decreased the expression of all genes analyzed in Region A (Fig. 4A), with one exception: the *Δ23040* mutant showed an expression pattern similar to the WT. This correlates with the result in Fig. 1A and B, where *Δ23040* retained its *in vitro* antibiotic ability. Thus, five Region B genes, except *23040,* appear to be essential for the expression of genes involved in rhizoviticin biosynthesis and cell lysis in Region A. We further examined how Region A genes respond to UV irradiation in both the WT and the *Δ22990* mutant (Fig. 4B). In the WT, all genes examined showed increased transcription after UV exposure, although the extent of upregulation varied. In contrast, UV-irradiated *Δ22990* cells maintained low levels of gene expression, although some genes showed UV-dependent increases. This confirms the requirement of *22 990* for the expression of Region A genes.

## Discussion

The antagonism of bacterial biocontrol agents against plant diseases has mostly been attributed to small antibiotics [19, 20, 33, 34]. In contrast, our study reveals a tailocin as a novel and distinct agent underlying the antagonistic activity of a biocontrol bacterium.

The R- and F-pyocins are well-characterized tailocins in *P. aeruginosa*, a Gram-negative bacterium belonging to the phylum *Pseudomonadota* [11, 15, 35]. The gene clusters responsible for their biosynthesis are juxtaposed in the genome and are thought to have evolved from a P2 (*Myoviridae*)-like and a lambda (*Siphoviridae*)-like prophage, respectively [36]. Syringacin and fluorescin are R-type tailocins in *Pseudomonas syringae* and *Pseudomonas fluorescens*, respectively [11, 15, 37]. Two different R-type tailocins have also been found in the plant-associated *Pseudomonas chlororaphis* [38]. Tailocins have also been discovered in other genera of *Pseudomonadota*, including *Stenotrophomonas maltophilia* (maltocin) [39], *Pectobacterium carotovorum* (carotovoricin) [40, 41], and *Xenorhabdus maltophilia* (xenorhabdicin) [42]. Moving to the phylum *Betaproteobacteria*, *Burkholderia cenocepacia* has a tailocin named BceTMilo [43]. Even in the Gram-positive phylum *Bacillota*, tailocins have been found in *Clostridium difficile* (diffocin), *Listeria monocytogenes* (monocin), and *Brevibacillus laterosporus* [44-46]. However, as far as our knowledge goes, no tailocins have been reported in the *Alphaproteobacteria* class. Rhizoviticin stands out as a rare example of an F-type tailocin, since most known tailocins are R-type, with the exception of F-pyocin in *Pseudomonas aerginosa* and monocin in *L. monocytogenes*.

The rhizoviticin-coding locus showed interesting genomic features. It consists of two distinct subregions, Regions A and B, which share significant homology with different prophages. Region B contains multiple genes typically found in the early regions of temperate phages, but a feature is not commonly seen in tailocin-coding loci. These genes include replication-related genes (*23000*; a Dna-like replication initiator, *23020*; a DnaB-like replicative DNA helicase) as well as the remnant of the integrase gene (*23140*). The exact origin of this chimeric structure remains uncertain, whether it resulted from integration of a preexisting chimeric phage into this locus or through genomic rearrangements involving two tandemly integrated prophages. However, the genes retained in Region B suggest that the rhizoviticin-coding locus represents an early stage in the evolutionary transition from a prophage to a complete tailocin-coding locus. In particular, at least five genes in Region B are critical for the expression of Region A genes responsible for rhizoviticin biosynthesis and cell lysis. Elucidation of the molecular mechanisms underlying this regulation is an important and challenging issue for future investigation.

An unexpected finding was that VAR03-1 mutants, lacking rhizoviticin production, exhibited a modest suppression of gall formation in plants. The underlying cause(s) of this residual antagonistic activity remains unknown, but may involve interbacterial antagonism via direct contact, such as toxin loading via the secretion system [17, 18, 47]. Another possibility is that the ability of VAR03-1 to colonize host plants contributes to its antagonistic behavior, affording a competitive advantage in nutrient acquisition and ecological niche occupation. Nevertheless, the essential role of rhizoviticin in the biocontrol activity of VAR03-1 is evident. The use of PCR-based detection targeting the rhizoviticin-coding locus will facilitate the isolation of additional rhizoviticin-producing bacteria. Furthermore, employing WGS to identify genomic regions similar to the rhizoviticin-coding locus can unveil potential biocontrol agents within the order *Rhizobiales* sourced from the rhizosphere of target crops. For the practical application of rhizoviticin-producing bacteria in plant disease biocontrol, several crucial aspects need to be addressed, including the spectrum of rhizoviticin and its receptor(s). The isolation of the rhizoviticin-insensitive VAT03-9 (Ti) may offer insights into the mechanism of rhizoviticin-binding to target cells. As our results have demonstrated, at least VAR03-1 is insensitive to rhizoviticin. This study is also critical in assessing the risk associated with the long-term use of VAR03-1 or rhizoviticin itself for crown gall biocontrol in the field due to the potential emergence of rhizoviticin-resistant pathogens. Additionally, exploring the variability of rhizoviticin, including the presence of subtypes with diverse spectra, as observed for R- and F-pyocins [36], could significantly enhance the versatility of rhizoviticin.

## Supplementary Material

Supplementary_Tables_230719_wrad003

Rhizoviticin_Supplementary_Figs_ISMEJ_231102_wrad003

## Data Availability

All data are included in the article and/or supporting information files. The WGS data of each bacterial isolate have been deposited in the DNA Data Bank of Japan (DDBJ) under accession numbers GCA_013427035.1 (VAR03-1), GCA_013426715.1 (VAT03-9 (Ti)), and GCA_013426735.1(VAT06-30).
