# Supplementary material for: Rhizoviticin is an alphaproteobacterial tailocin that mediates biocontrol of grapevine crown gall disease
Source: ISME J. 2024 Jan 18;18(1):wrad003. doi: 10.1093/ismejo/wrad003 (PMC10811719; doi:10.1093/ismejo/wrad003)
Supplement: Rhizoviticin_Supplementary_Figs_ISMEJ_231102_wrad003 [file rhizoviticin_supplementary_figs_ismej_231102_wrad003.pdf]

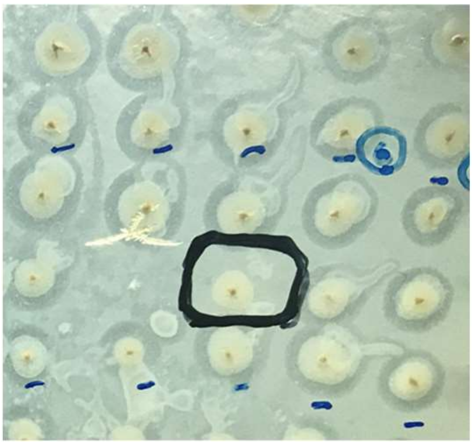

Fig. S1 Screening of the antagonism-deficient mutants of *A. vitis* VAR03-1 against *A. vitis* VAT03-9 (Ti).
The colonies of the transposon-insertion mutants of VAR03-1 were spotted on duplicated agar plates. After overnight incubation, soft agar medium supplemented with *A. vitis* VAT03-9 (Ti) was overlaid. Halo-less colonies were then screened, as shown in the picture surrounded by a black line. Finally, 4 mutants were obtained from about 4,000 colonies.

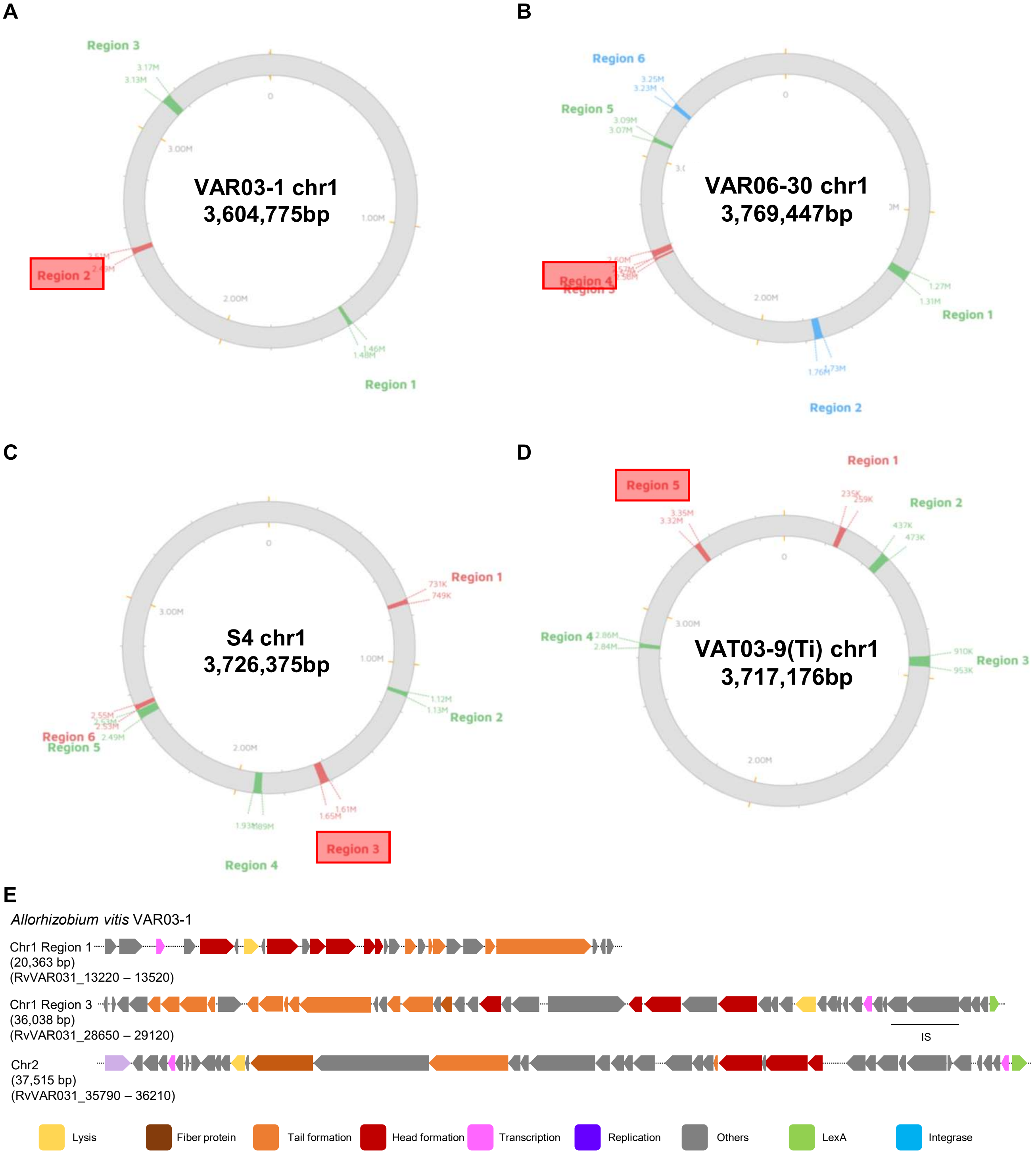

Fig. S2 Positions of the predicted prophage or prophage-like regions on Chr 1 of *A. vitis* VAR03-1, *A. vitis* VAR06-30, *A. ampelinum* S4, and *A. vitis* VAT03-9 (Ti) and the genetic organization of three prophages in VAR03-1.
A-D The positions of the predicted prophage or prophage-like regions in VAR03-1 (AP023268.1) (**A**), VAR06-30 (a non-antagonistic and pathogenic strain) (AP023272.1) (**B**), S4 (a pathogenic strain) (CP000633.1) (**C**), and VAT03-9 (Ti) (a pathogenic strain) (AP023279.1) (**D**) detected by the PHASTER program are shown. Region 2 of VAR03-1 corresponds to the rhizoviticin-coding region. Region 4 in VAR06-30, Region 3 in S4, and Region 5 in VAT03-9 (Ti) were the prophages showing sequence similarity to Region A of the rhizoviticin-coding region indicated in Fig 1. **E** The gene organizations of three prophage or prophage-like regions in Chr 1 and Chr 2 of VAR03-1 other than the rhizoviticin-coding region. Each gene is represented by arrows with colors based on its potential functions. Region 3 in Chr 1 and the prophage in Chr 2 are potentially intact prophages, as they appear to contain a complete set of genes for phage functions.

A

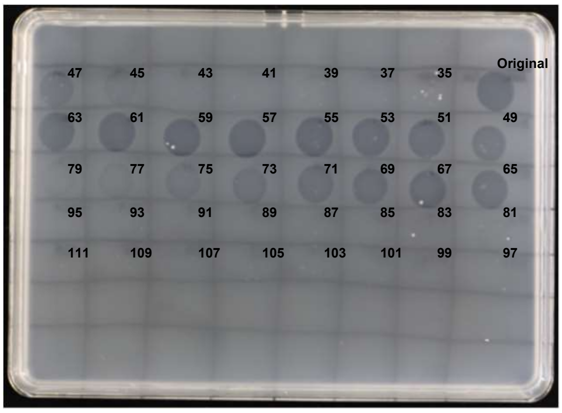

B

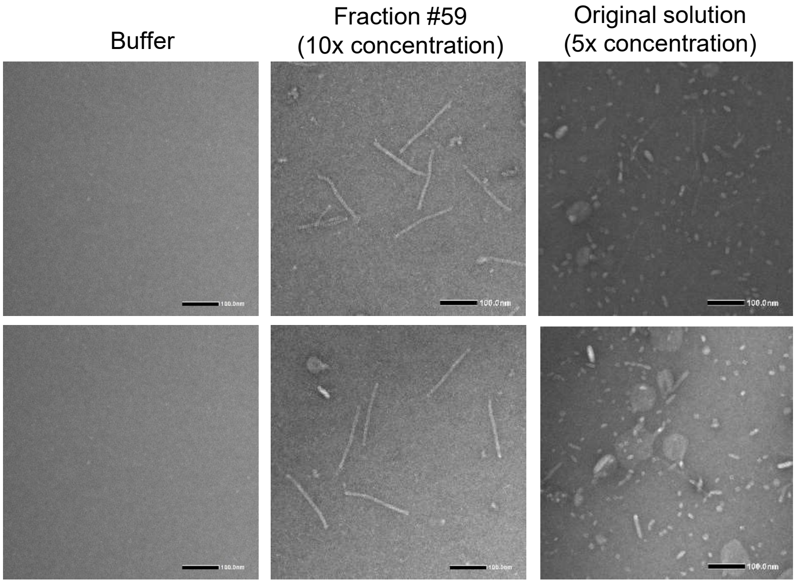

Fig. S3 Purification of rhizoviticin.

A Bioassay for antibiotic activity of each fraction obtained by gel filtration chromatography. The culture filtrate of *Allorhizobium vitis* VAR03-1 was ultracentrifuged after precipitation with polyethylene glycol. The precipitate was resuspended in the buffer and fractionated by gel filtration chromatography using a HiPrep 16/60 Sephacryl S-500 HR column at an elution rate of 0.7 ml/min. The fractions (2 ml each) corresponding to an elution volume of 35–111 ml were subjected to the assay. Ten µl of each fraction and the original solution were spotted on agar medium containing *A. vitis* VAT03-9 (Ti) and incubated overnight to evaluate the growth inhibitory activities of each fraction. The number represents the elution volume (in milliliters). **B** Morphological examination of the fractionated samples by transmission electron microscopy (TEM). The 10-fold concentrated fraction #59, the 5-fold concentrated original solution, and the buffer were stabilized on the super support film-coated copper grid and stained with uranyl acetate. The phage tail-like particles were observed only in fraction #59, which showed antibiotic activity. Bar, 100 nm.

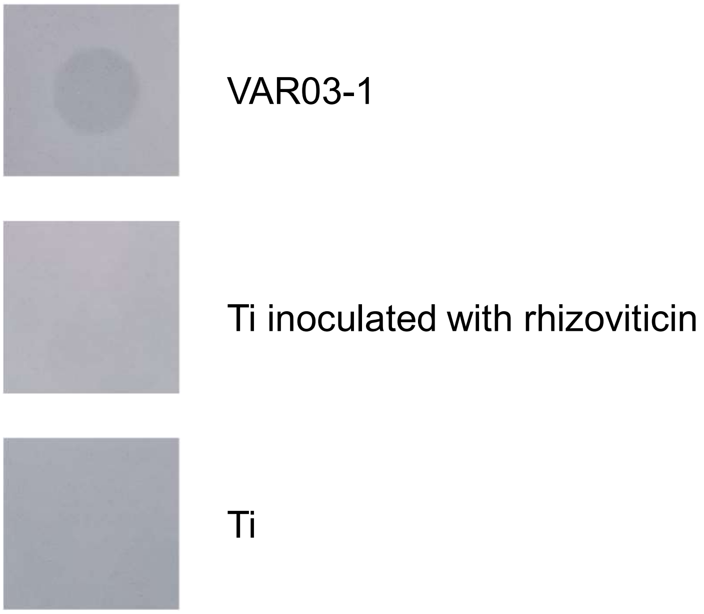

Fig. S4 Non-self-replication of the killing activity in the culture filtrate of *A. vitis* VAR03-1 against *A. vitis* VAT03-9 (Ti).

(*Top*) Halo formation by the spot of the 10-fold concentrated and size (100k) fractionated culture filtrate of VAR03-1 on soft agar containing VAT03-9 (Ti). (*Middle and Bottom*) No growth inhibitory activities by the droplets of the 10-fold concentrated and size (100k) fractionated supernatant of the overnight culture of VAT03-9 (Ti) inoculated with (middle) or without (bottom) one microliter of the concentrated culture filtrate of VAT03-1 on soft agar containing VAT03-9 (Ti).

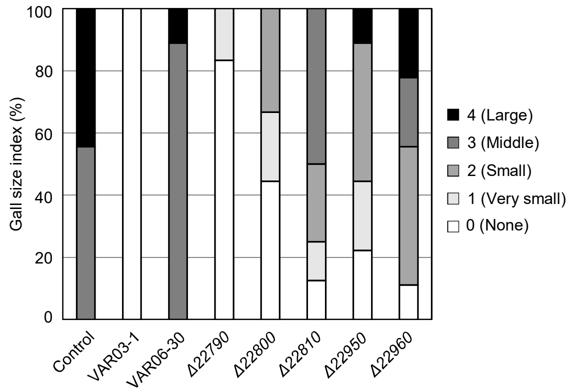

Fig. S5 Reduced anti-tumorigenic activities in rhizoviticin-deficient mutants.

The levels of tumor formation on tomato by *Allorhizobium vitis* VAT03-9 (Ti) when coinoculated with *A. vitis* VAR06-30, *A. vitis* VAR03-1, or rhizoviticin-deficient mutants of VAR03-1 are shown. The indicated strains and mutants were grown in liquid medium and the cultures whose OD600 was adjusted to 0.1 with fresh medium, were inoculated with VAT03-9 (Ti) on tomato stems using toothpicks. After three weeks, the tumors formed at 8 inoculation points were classified into 5 categories according to size, as indicated, and the proportion of each category was presented as a disease index for each strain/mutant. Experiments were repeated three times, and representative results are shown.

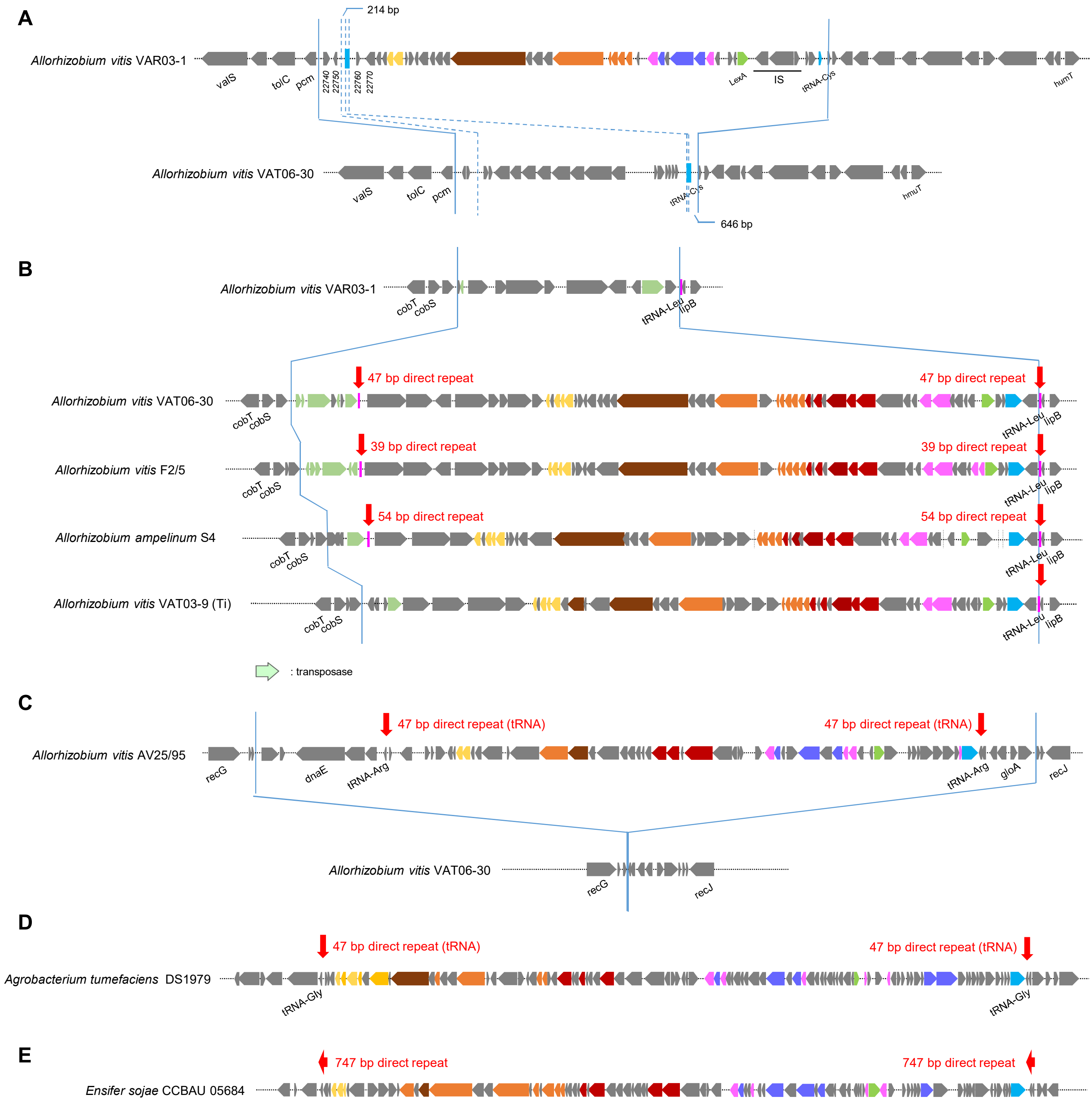

Fig. S6 Genomic positions and potential *att* sequences of the rhizoviticin-coding region and related prophages. **A** The genomic organization of the rhizoviticin-coding region and its flanking regions in *Allorhizobium vitis* VAR03-1. The genomic organization of the corresponding genomic region in *A. vitis* VAR06-30 is also shown. It appears that an ancestral prophage of the rhizoviticin-coding region was inserted between a tRNA-Cys gene and a short region (646 bp) indicated light blue, most probably into the 3' end of the tRNA-Cys gene. No direct repeat sequences corresponding to *attL*/*R* sites were found at either end of the rhizoviticin-coding region. In *A. vitis* VAR06-30, a 14.7-kbp segment has been inserted next to the region indicated in light blue. **B** The genomic organization of the prophage containing a segment homologous to Region A in the rhizoviticin-coding region in *A. vitis* VAR06-30, VAT03-9 (Ti) and F2/5 and *A. ampelinum* S4. The genomic organization of the corresponding genomic region in *A. vitis* VAR03-1 is also shown. The prophages are apparently integrated into the 3' end of the tRNA-Leu gene. Direct repeat sequences of 39-51 bp were found for three prophages, which (or parts of them) represent the *attL/R* sites. In VAR03-1, a 14.3 kbp segment was inserted into this locus. **C** The genomic organization of a prophage in *A. vitis* AV25/95 containing a segment homologous to Region B in the rhizoviticin-coding region. The genomic organization of the corresponding genomic region in *A. vitis* VAR06-30 is also shown. The prophage appear to be inserted into a tRNA-Arg gene with 47 bp *att* sequences. This prophage is located between *recG* and *recJ*, but the left and right flanking regions are missing in VAR06-30. **D, E** The genomic organization of the prophages in *A. tumefaciens* DS1979 (**D**) and *Ensifer sojae* CCBAU 05684 (**E**), both of which contained a segment homologous to Region B in the rhizoviticin-coding region. The prophage of DS1979 has been inserted into the 3' end of the tRNA-Gly gene with 47 bp of *att* sequences. In CCBAU 05684, the prophage region was flanked by 747 bp direct repeat sequences, but it is unknown whether this repeat sequence serves as the *att* sequence for this phage.

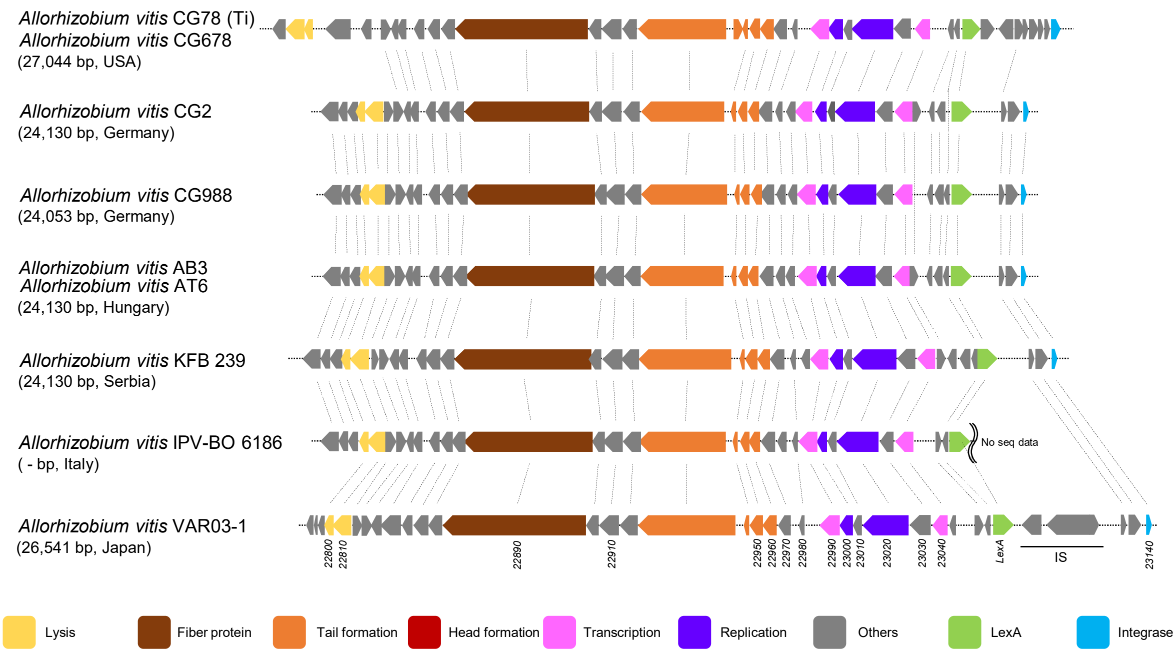

Fig. S7 Schematic representation of the genomic regions in eight *A. vitis* strains that are very similar to the rhizoviticin-coding region of *A. vitis* VAR03-1.

The genetic organization of each genomic region found in strains KFB 239 (VOLI01000001.1), CG78 (WPIA01000023.1), CG678 (CP056042.1), IPV-BO 6186 (VOLK01000003.1), AB3 (MAVS02000002.1), AT6 (MBEX02000008.1), CG988 (WPHQ01000002.1), and CG2 (JABAEI010000004.1) are shown. Each gene is represented by arrows with colors based on their potential functions and homologous genes are connected by dotted lines. For each region, the total length and the countries where each strain was isolated are shown. All regions are located at the same genomic position as the rhizoviticin-coding region of VAR03-1.
